# Functional characterization of *Francisella tularensis* subspecies *holarctica* genotypes during tick cell and macrophage infections using a proteogenomic approach

**DOI:** 10.3389/fcimb.2024.1355113

**Published:** 2024-03-04

**Authors:** Sara Doina Schütz, Maximilian Brackmann, Nicole Liechti, Michel Moser, Matthias Wittwer, Rémy Bruggmann

**Affiliations:** ^1^ Interfaculty Bioinformatics Unit, University of Bern and Swiss Institute of Bioinformatics, Bern, Switzerland; ^2^ Spiez Laboratory, Federal Office for Civil Protection, Spiez, Switzerland; ^3^ Graduate School for Cellular and Biomedical Sciences, University of Bern, Bern, Switzerland

**Keywords:** Francisella, tularemia, tick cell, macrophages, RNA-seq, LC-MS/MS, transcriptomics, proteomics

## Abstract

Tularemia is a vector-borne disease caused by the Gram-negative bacterium *Francisella tularensis*. Known hosts and vectors in Europe are hare and ticks. *F. tularensis* is transmitted from ticks and animals, but also from the hydrotelluric environment and the consumption of contaminated water or food. A changing climate expands the range in which ticks can live and consequently might contribute to increasing case numbers of tularemia. Two subspecies of *F. tularensis* are human pathogenic. *Francisella tularensis tularensis (Ftt)* is endemic in North America, while *Francisella tularensis holarctica (Fth)* is the only subspecies causing tularemia in Europe. *Ft* is classified as a category A bioterrorism agent due to its low infectious dose, multiple modes of transmission, high infectivity and potential for airborne transmission and has become a global public health concern. In line with the European survey and previous phylogenetic studies, Switzerland shows the co-distribution of B.6 and B.12 strains with different geographical distribution and prevalence within the country. To establish itself in different host environments of ticks and mammals, *F. tularensis* presumably undergoes substantial changes on the transcriptomics and proteomic level. Here we investigate the transcriptomic and proteomic differences of five strains of *Fth* upon infection of rabbit macrophages and tick cells.

## 1 Introduction

*Francisella tularensis (Ft)*, a Gram-negative coccobacillus, is the causative agent of the zoonotic disease tularemia affecting various species. The severity and clinical manifestation of tularemia vary depending on the route of infection and subspecies involved. Of particular importance are the subspecies *Ft tularensis* and *Ft holarctica*, which can cause tularemia in humans (Tärnvik and Berglund, 2003). *Francisella tularensis* subsp. *tularensis (Ftt)* is the most virulent pathogen that can cause fatal pneumonia in humans with only a few aerosol-transmitted bacteria. In contrast, the subspecies *Ft holarctica (Fth)* is less virulent in humans (Kingry and Petersen, 2014). *Francisella novicida (Ftn)* was originally classified as a third subspecies of *Ft* but is now recognized as a separate species. *Ftn* mainly infects immunocompromised or elderly people who are exposed to environmental sources (Kingry and Petersen, 2014; Chua et al., 2021). Advances in whole genome sequencing and canonical single nucleotide polymorphism (canSNP) analysis have revealed distinct phylogenetic clades within the subspecies *Fth* despite its small genome and limited genetic diversity. The major basal clades identified in this subspecies are B.4, B.6, B.12 and B.16, which can be further subdivided into subclades. Clade B.12 and subclade B.6 are both found in Germany and Switzerland (Dwibedi et al., 2016). In Switzerland, all *Fth* isolates isolated and sequenced in the last 8 years belonged to the basal clade B.6, with none belonging to the previously identified B.12 clade at the Swiss-German border. Different geographical distributions and host preferences were observed for the different B.6 subclades. While the most widespread subclade B.45 is distributed throughout northern Switzerland and was found in humans, animals and ticks, the B.6 subclades B.86 and B.46 are narrowly distributed and could only be isolated in human and animal sources (Dwibedi et al., 2016; Wittwer et al., 2018; Schütz et al., 2023). Histopathological examinations have revealed considerable differences in pathogenicity between strains belonging to the B.6 and B.12 clade. Infections caused by B.12 strains were characterized by polyserositis affecting the kidneys, pleura and pericardium. Conversely, rabbits infected with B.6 consistently showed splenitis and hepatitis as prominent histopathologic features (Origgi and Pilo, 2016; Kreizinger et al., 2017). These results are consistent with experimental studies in rats, which showed significant differences in weight loss, mortality rate and time to recovery between the two basal clades (Kreizinger et al., 2017).

The pathogenicity of *Francisella* is related to its ability to multiply in the cytosol of phagocytes, such as macrophages or dendritic cells. After phagocytosis, the bacteria briefly reside in a membrane-bound phagosome, but then break through the phagosomal membrane and escape into the cytosol of the host cell, where they multiply (Brodmann et al., 2017). *Francisella* modulates virulence through complex regulatory processes involving molecular signaling, gene transcription, translation and post-translational modifications. Pathogenicity is based on generating virulence factors that target host cells and suppress the immune response (Spidlova et al., 2020). The major virulence factors of *Ft* include the capsule (Su et al., 2007), the lipopolysaccharide layer on the surface (Ancuta et al., 1996; Miller et al., 2005; Wang et al., 2007), membrane vesicles (Golovliov et al., 2003; Klimentova et al., 2019), and secretion systems, in particular the type VI secretion system (T6SS) (Lindgren et al., 2004; Nano et al., 2004; De Bruin et al., 2007; Bröms et al., 2010). T6SS includes proteins encoded by *Francisella* pathogenicity island (FPI) genes such as *pdpABCDE*, *iglABCDEFGHIJ*, *vgrG* and domain-containing protein (*dotU*) (Nano et al., 2004; De Bruin et al., 2007; Bröms et al., 2010). FPI proteins play a critical role in *Francisella* intracellular replication, and mutant strains lacking at least one FPI gene exhibit virulence defects *in vivo* (Santic et al., 2005). While some FPI proteins, such as IglG, may not be vital for intracellular growth, their absence can lead to delayed phagosomal escape (Bröms et al., 2011). To unravel the pathogenesis of *Ft*, a comprehensive understanding of this process, including the MglA/SspA/PigR complex and other regulatory proteins that control the transcription of virulence genes, is essential (Lauriano et al., 2004; Brotcke et al., 2006; Guina et al., 2007; Charity et al., 2009; Dai et al., 2011).

While *Fth* has a wide distribution across North America, Eurasia and even Australia (Aravena-Román et al., 2015; Dantas-Torres, 2015), its ecological associations vary in different regions. In northern Sweden and Finland, this subspecies is often associated with blood-sucking mosquitoes in areas near watercourses, while in central Europe it is more commonly associated with ticks (Rydén et al., 2012; Rossow et al., 2014). In Switzerland, ticks are thought to be the main vectors of tularemia. Climatic changes could favor the spread of ticks and consequently also the prevalence of the pathogen (Jongejan and Uilenberg, 2004; Dantas-Torres, 2015)015; Jongejan and Uilenberg, 2004). Tick-borne pathogens must be efficiently transmitted between mammalian and arthropod hosts after the tick bite (Galletti et al., 2016; Murfin et al., 2019). During the transition from mammalian host to tick vector, *Ft* likely undergoes significant metabolic and transcriptional changes. In other tick-borne diseases, such as *Borrelia burgdorferi*, the causative agent of Lyme disease, factors such as temperature and pH are known to be critical factors in the transition between mammalian host and tick. These factors lead to changes in bacterial surface proteins that facilitate the uptake of *B. burgdorferi* by ticks (Carroll et al., 2000; Brooks et al., 2003). These two well-characterized surface-exposed lipoproteins, OspA and OspC, are differentially expressed in *B. burgdorferi* in response to these environmental cues (Pal et al., 2004; Yang et al., 2004).

Due to the challenges and biosafety concerns of working with infected ticks, tick cell lines offer an alternative to study factors of bacterial infection of arthropods. Recent technological advances in mass spectrometry and next-generation sequencing have transformed our ability to systematically study complex biological phenomena. Especially, liquid chromatography-coupled mass spectrometry (LC-MS/MS) in data-independent acquisition (DIA) mode has greatly enhanced our capabilities for comprehensive proteogenomic analyses and allows more accurate comparative analyses of protein expression profiles.

While previous studies have shed light on how *Ft* responds to environmental stimuli and how these changes enable tick infection, there are still significant gaps in knowledge regarding the adaptation of different *Ft* subspecies to the tick environment. Two questions were addressed in this study: (i) To better understand the adaptation of *Fth* during tick-to-mammalian host transmission, the expression of genes in tick cells and rabbit macrophage were compared. (ii) To identify differences in the pathogenicity of the *Fth* basal clades B.6 and B.12, a combination of proteomic and transcriptional analyses was performed.

## 2 Methods

### 2.1 Cell culture

IRE/CTVM19 ticks derived from the embryonic stage of *Ixodes ricinus* were kindly provided by L. Bell-Sakyi (Tick Cell Biobank, University of Liverpool, England) and maintained at 28°C as previously described (Bell-Sakyi et al., 2007). The cell line was grown in flat Nunclon tubes (Merck, Darmstadt, Germany) in 2.2 ml Leibovitz’s L-15 medium (Thermo Fisher Scientific, Massachusetts, USA) supplemented with 20% fetal bovine serum, 10% tryptose phosphate broth, 2 mM L-glutamine, penicillin (100 U/ml) and streptomycin (100 μg/ml) (Thermo Fisher Scientific, Massachusetts, USA). Rabbit bone marrow macrophages (RBMDM) obtained from the tibia or femur of New Zealand White Rabbits (CellBiologics, Chicago, USA) were cultured according to the manufacturer’s recommendations. Cells were incubated at 37°C and 5% CO_2_ in the recommended macrophage media supplement kit containing basal medium with murine macrophage growth supplement, antibiotics and fetal bovine serum (CellBiologics, Chicago, USA).

### 2.2 Infection of IRE/CTVM19 and RBMDM with *Fth* genotypes and total RNA and protein extraction

The strains used in this study Ft10, Ft17, Ft29, Ft38, Ft39, representing subclades, B.86 (B.6), B.46 (B.6), B.33 (B.12), B.50 (B.6) and B.51 (B.6) (**Supplementary Table 1**), were cultured on Polyvitex^®^ agar plates (bioMérieux, Marcy-l’Étoile, France) at 37°C and 5% CO_2_ overnight. 3x10^5^ IRE/CTVM19 tick cells were seeded in 1 ml and maintained at 28°C, while 8x10^5^ RBMDM cells were seeded in 1 ml and incubated at 37°C for the duration of the experiment. The host cells were infected with *Fth* strains with a multiplicity of infection (MOI) of 100 in a reduced medium volume of 0.5 ml medium, followed by a centrifugation step of the plate for 5 minutes at 500 g. Infected cultures were incubated for one hour. The medium was replaced with medium containing 10 mg/ml gentamicin (Thermo Fisher Scientific, Massachusetts, USA) and incubated for another hour to remove extracellular bacteria and prevent reinfection. The medium was then replaced with gentamicin-free medium and the infected cells were incubated for a further 20 hours. As control, *Fth* strains were cultured in tick cell medium for 20 hours at 28°C. Growth of *Fth* in macrophage cells, tick cells and tick cell medium was monitored by quantitative PCR at different time points. For RNA extraction, the medium was removed, the cells were washed twice with medium before the cells were lysed with QIAzol (QIAGEN, Hilden, Germany).

### 2.3 RNA sequencing and data analysis

Total RNA of the five *Fth* strains grown in triplicate in tick cells, macrophages and tick cell medium was extracted using the RNeasy Mini Kit (QIAGEN, Hilden, Germany). After removal of rRNA with the Illumina Ribo-Zero Plus rRNA Depletion Kit (Illumina, San Diego, USA), the TruSeq Stranded mRNA Library Prep Kit (Illumina, San Diego, USA) with 1 μg total RNA was used to construct sequencing libraries. The fragment size of the libraries was confirmed using the Agilent 2100 Bioanalyzer (Agilent, Santa Clara, USA). One sample (Ft10_medium_1) was excluded due to high RNA degradation. The libraries were sequenced on an Illumina NovaSeq 6000 in paired-end mode (50-bp reads). The quality of the raw data was assessed using FastQC (version 0.12.1) (Simon Andrews, n.d). The raw data were mapped to *Fth* FTNF002-00 (ASM1778v1) sequence using Bowtie2 (version 2.5.2) (Langmead and Salzberg, 2012). Mapped reads per CDS were summarized in a count-matrix using FeatureCount (version 2.16.0) (Liao et al., 2014) using the FTNF002-00 annotation. Differential gene expression analysis was performed using DEseq2 (version 1.42.0) (Love et al., 2014) using an adjusted p-value < 0.05(padj) and absolute Log2 fold change (LFC) > 1. The R function pheatmap was used to draw clustered heatmaps (version 1.0.12) (GitHub - Raivokolde/Pheatmap: Pretty Heatmaps, n.d). Over-representative analysis (ORA) and Gene Set Enrichment Analysis (GSEA) was performed using the R package clusterProfiler (version 4.10.0) (Wu et al., 2021)based on the Kyoto Encyclopedia of Genes and Genomes (KEGG) pathway annotations.

### 2.4 Protein sample preparation, LC-MS/MS analysis and data analysis

Proteins from cell lysates in QIAzol were extracted using an adapted one-pot protocol for solid-phase enhanced sample preparation (SP3) (Hughes et al., 2018) on a KingFisher Apex (Thermo Fisher Scientific, Massachusetts, USA). 25 ug of protein was used as starting material and the proteins were not reduced or alkylated. Protein digestion was performed using the Rapid-Digestion Trypsin/Lys-C Kit (Promega, Wisconsin, USA) according to the manufacturer’s recommendations.

Peptide samples were separated on a 15 cm C18 reversed-phase column (Aurora Elite CSI, IonOpticks, Australia) using a Bruker nanoElute. The gradient elution increased from 2% B to 35% B in 25 minutes, then from 35% B to 95% B in 0.5 minutes and remained at 95% B for 5.5 minutes with a flow rate of 300 nl/min. The column was kept at 50°C. Data were acquired using a timsTOF Pro 2 (Bruker, Bremen, Germany) in dia-PASEF mode with customized DIA windows optimized with py_diAid (version 0.0.17) (Skowronek et al., 2022). Data were analyzed using DIA-NN with the proteome of FTNF002-00 (Uniprot accession UP000000261) as database for *in silico* library prediction (DIA-NN version 1.8.2 beta) (Demichev et al., 2019). DIA-NN library was generated with strict trypsin specificity (KR not P), allowing for a single failed cleavage site. Precursor range and fragment ion range was set to 150 - 1700 m/z. Peptides with 7-30 amino acids in length and charge states of 2 - 4 were considered. N-terminal methionine excision was allowed as variable modification and option match-between-runs was enabled. False discovery rate threshold was set to 0.01. Quantification of proteins was performed using QuantUMS algorithm (Kistner et al., 2023) and results were analyzed with the R package tidyproteomics (version 1.5.10) (Jones et al., 2023).

## 3 Results

### 3.1 *Fth* changes the transcription profile depending on the environment

RNA sequencing of the 45 isolates analyzed in this study yielded an average of 48,872,979 raw reads per isolate. The *Fth* isolates grown in medium conditions have an average total number of 32,814,935 reads mapped to the FTN002-00 reference. 31% of the alignments were successfully assigned to the CDS on average. The macrophage had 86,264 alignments with 64% and the tick 11,340,353 with 78%. The data is available in BioProject accession number PRJNA1050617.

**Figure 1:**
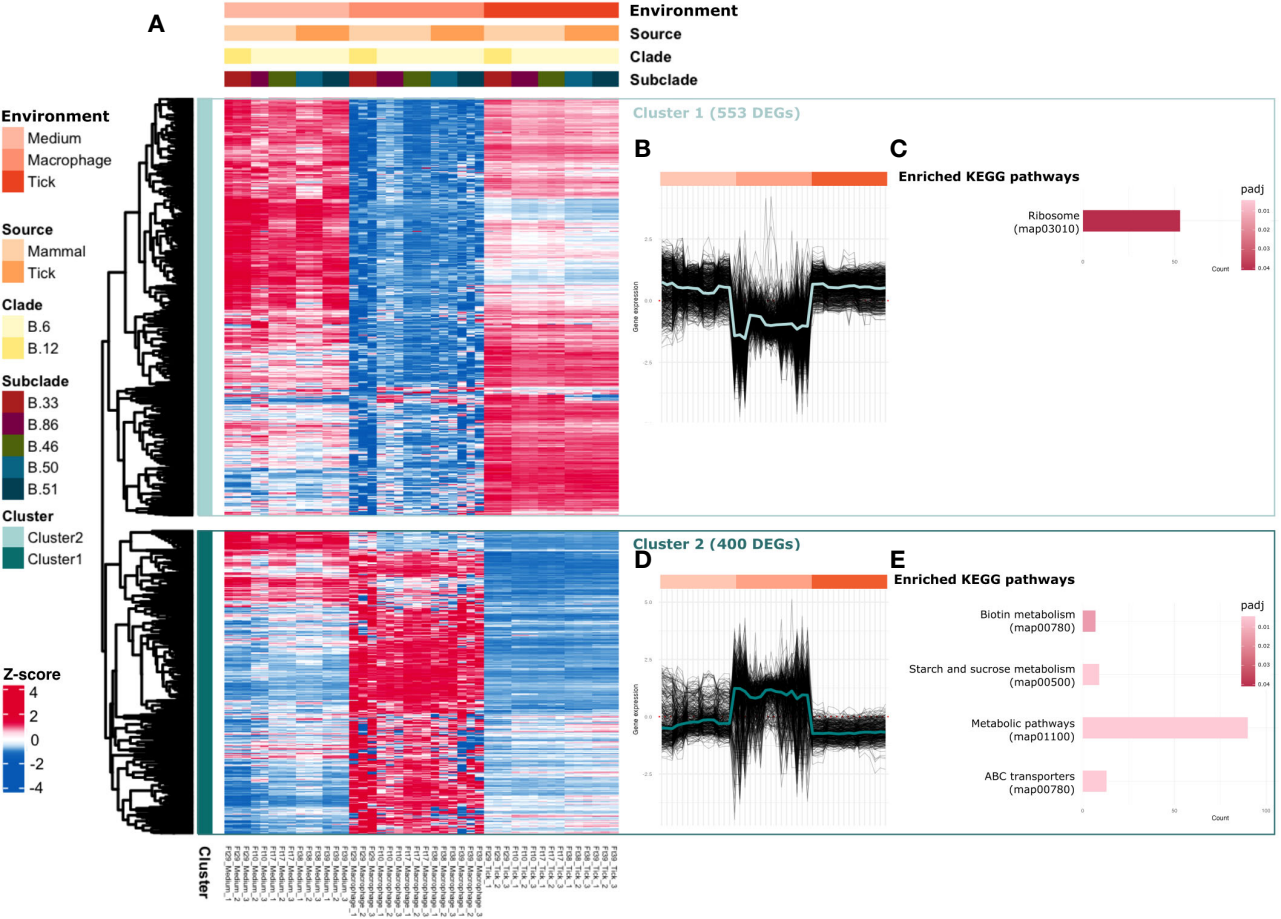
*Fth alters the transcriptional profile depending on the environment.* Heatmap **(A)** shows the VST values of the DEGs (padj < 0.05, absolute LFC > 1), cluster profiles of Cluster 1 **(B)** and Cluster 2 **(D)** determined by hierarchical cluster analysis are shown next to the heatmap. ORA of enriched KEGG pathways of the DEGs of cluster 1 **(C)** and cluster 2 **(E)** are shown next to it.

RNA-seq analysis showed that the expression profiles of all *Fth* strains clustered according to the different environments (medium, macrophage or tick), strongly suggesting that *Fth* changes its transcriptional profile depending on its host. Of the 2084 annotated genes, 953 were found to be differentially expressed (padj < 0.05, absolute LFC > 1) when comparing *Fth* grown in the tick with those grown in the macrophage. The heatmap in **Figure 1A** shows the gene expression of the five *Fth* strains representing subclades B.33, B.86, B.46, B.50 and B.51 in all three environments of these differentially expressed genes (DEGs). Hierarchical cluster analysis of DEGs revealed two distinct clusters. Cluster 1, consisting of 553 DEGs, showed upregulation under macrophage cell infection conditions and maintained comparable expression levels under tick cell infection compared to medium (**Figure 1B**). ORA showed significant enrichment in the ribosomal pathway (**Figure 1C**). Cluster 2, comprising 400 DEGs, showed a downregulated profile under macrophage cell infection but maintained equal expression levels under tick cell infection compared to medium (**Figure 1D**). ORA identified significant enrichment in four KEGG metabolic pathways, including biotin metabolism (essential for bacterial growth and survival), starch and sucrose metabolism (involving enzymatic processes in energy storage and utilization), metabolic pathways, and ABC transporters (a family of membrane proteins that facilitate the transport of various substances across biological membranes) (**Figure 1E**).

### 3.2 KEGG pathway enrichment analysis of DEGs between the tick cell and macrophage environment

**Figure 2:**
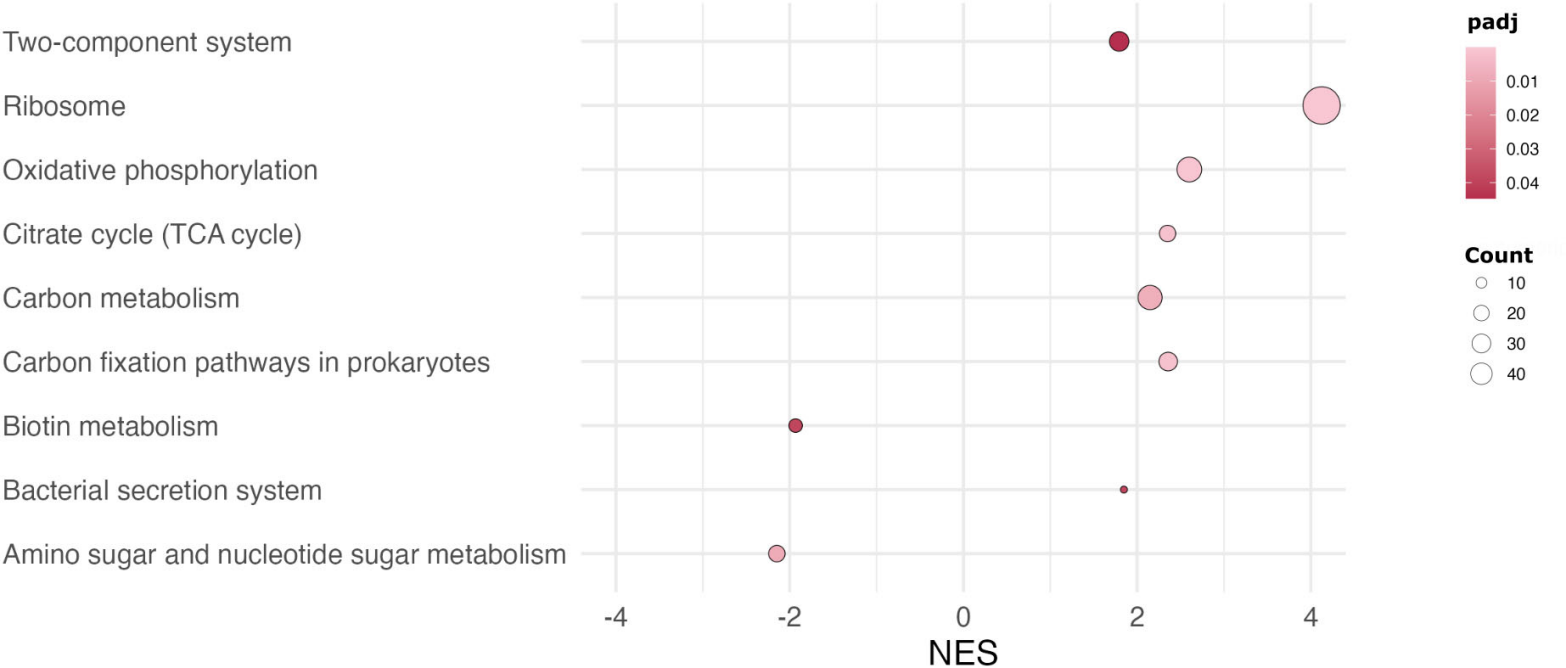
*KEGG pathway enrichment analysis of DEGs between the tick and macrophage environment.* Dot plot shows identified KEGG pathways by GSEA analysis (padj < 0.05, absolute LCF > 1) in tick versus macrophage environment. The size of the circles represents the number of genes in each pathway (Count); the colors represent the negative logarithms of the adjusted p-values (padj); NES: normalized enrichment score.

GSEA of DEGs revealed differential regulation of KEGG pathways in tick and macrophage environments. Several enriched pathways were involved in metabolism, energy production, protein transport across membranes and signaling pathways. These included “Ribosome”, “Oxidative phosphorylation”, “Carbon fixation pathways in prokaryotes”, “TCA cycle”, “Carbon metabolism”, “Bacterial secretion system”, “Two-component system”. It is noteworthy that two metabolic pathways related to the metabolism of amino sugars and nucleotide sugars and biotin were downregulated in this context (**Figure 2**). This suggests that *Fth* is dependent on the host cell for the provision of secondary metabolites and consequently downregulates the systems for their synthesis.

### 3.3 Known transcription factors and their targets involved in virulence are regulated differently in the tick cell and macrophage environment

**Figure 3:**
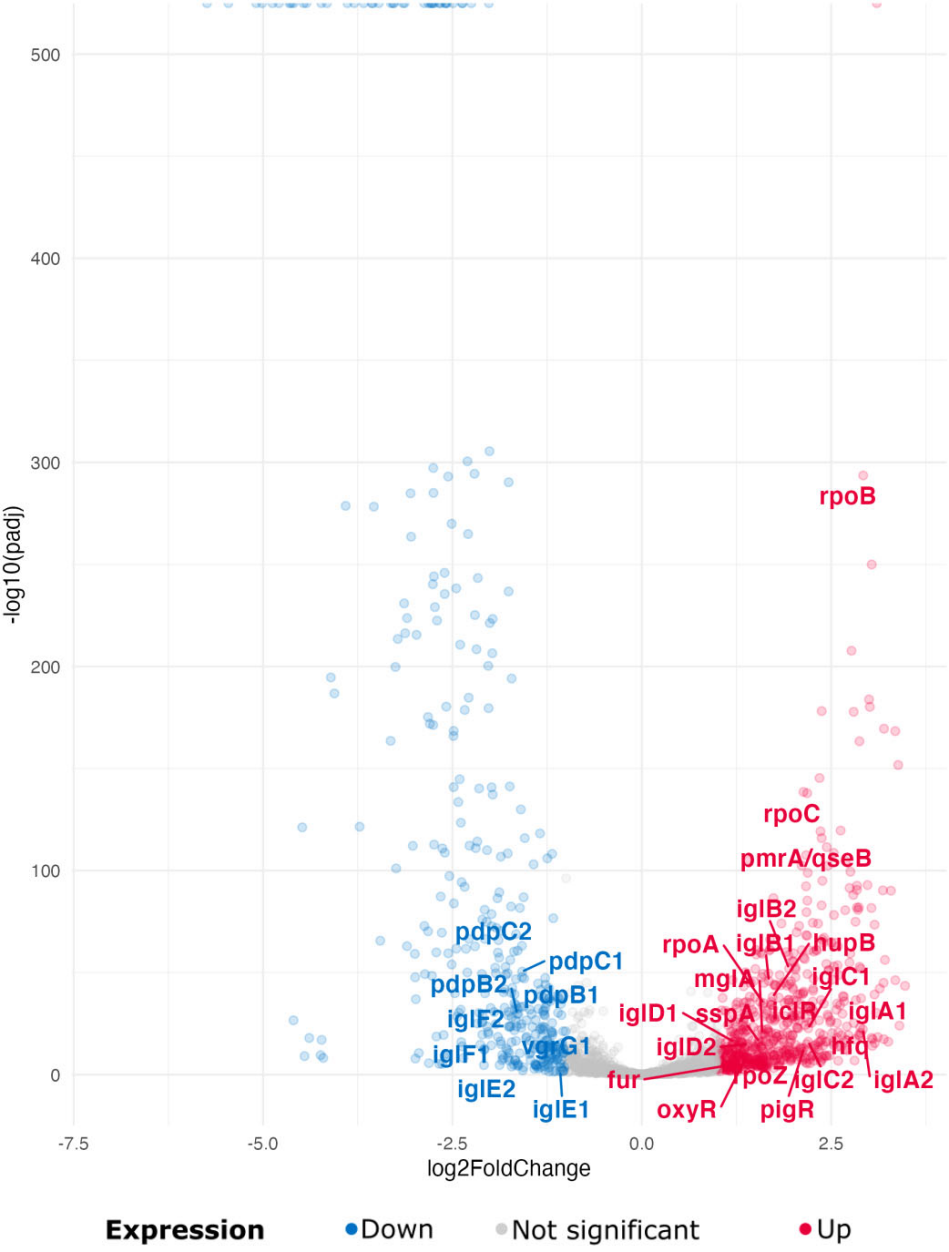
Known transcription factors and their targets involved in virulence are differentially expressed between the tick and macrophage environments. Volcano diagram depicting selected differentially expressed transcription factors and their regulated targets in tick and macrophage environments. Bottom (blue): LFC < -1, padj < 0.05; Not significant (gray): LFC < 1 and > -1, padj > 0.05; Up (red): LFC > 1, padj < 0.05.

There is a lack of understanding of the factors and molecular mechanisms that control *Francisella* pathogenicity. The transcriptional regulatory system mediating FPI activation has been described in recent studies. These known key transcription factors involved in pathogen virulence and their regulated FPI targets are differentially regulated in this study. They are shown in volcano plots for the tick and macrophage environments (**Figure 3**) and detailed in **Table 1**.

**Table 1:** Differentially expressed general transcriptional regulators and their regulated targets for the tick vs macrophage, macrophage vs medium and tick vs medium conditions (padj < 0.05); LFC, Log2 fold change; padj, adjusted p-value.

Gene_ID	Gene	Protein_ID	Description	Tick vs Macrophage		Macrophage vs Medium		Tick vs Medium	
				LFC	padj	LFC	padj	LFC	padj
FTA_RS06035	mglA	WP_003016250.1	interacting with SspA, regulation of FPI genes, necessary for intracellular replication	1.59	1.28E-20	-2.03	5.02E-33	-0.45	6.43E-38
FTA_RS08125	sspA	WP_003019455.1	interacting with MglA, regulation of FPI genes, necessary for stress response	1.55	8.65E-17	-2.26	3.57E-34	-0.71	1.22E-120
FTA_RS02370	pigR	WP_003017929.1	interacting with MglA/SspA, regulation of FPI genes	2.14	1.26E-13	-6.66	9.08E-121	-4.52	0.00E+00
FTA_RS04605	hupB	WP_003015647.1	global regulator, DNA non-specific binding protein	1.73	7.68E-39	-4.48	1.57E-251	-2.76	8.90E-265
FTA_RS04620	hfq	WP_003015654.1	RNA-binding protein, necessary for stress response, negative regulator of pdp operon	2.35	4.97E-21	-4.71	1.36E-80	-2.36	0.00E+00
FTA_RS06925	iclR	WP_003016591.1	important only for F. novicida virulence in vivo	1.99	5.00E-20	-2.87	2.61E-40	-0.88	6.38E-124
FTA_RS05170	oxyR	WP_003015875.1	regulation of genes involved in oxidative stress response	1.38	2.33E-09	-1.39	1.28E-09	NA	NA
FTA_RS09300	fur	WP_003017396.1	regulation of iron concentration	1.11	4.16E-05	-2.22	6.19E-17	-1.12	7.04E-221
FTA_RS01380	rpoA	WP_003021582.1	RNAP subunit alpha, gene transcription	1.61	9.25E-36	-2.02	6.47E-56	-0.42	4.33E-25
FTA_RS08830	rpoB	WP_010032708.1	RNAP subunit β, gene transcription	2.92	2.48E-294	-2.67	3.30E-244	0.25	1.19E-10
FTA_RS08825	rpoC	WP_003019910.1	RNAP subunit β’, gene transcription	2.18	1.17E-138	-2.98	2.55E-256	-0.8	7.16E-98
FTA_RS07775	rpoZ	WP_003019409.1	RNAP subunit ω, gene transcription	1.9	5.50E-10	-3.68	5.56E-34	-1.78	0.00E+00
FTA_RS05365	rpoD	WP_003015963.1	sigma factor 70, major sigma factor	0.14	3.97E-02	0.92	3.21E-44	1.06	0.00E+00
FTA_RS04385	rpoH	WP_003015458.1	sigma factor 32, regulation of heat shock proteins	0.24	1.95E-03	-0.41	4.49E-08	-0.18	4.70E-07
FTA_RS02900	pmrA/qseB	WP_010032924.1	response regulator, important for biofilm formation in F. novicida, intracellular replication, interacting with MglA/SspA	2.37	1.23E-116	-1.75	2.92E-63	0.63	4.29E-40
FTA_RS00630	pdpA1	WP_003016195.1	FPI, Pathogenicity determinant protein A1	NA	NA	-3.06	1.09E-60	-3.37	0.00E+00
FTA_RS05935	pdpA2	WP_003016195.1	FPI, Pathogenicity determinant protein A2	NA	NA	-3.02	1.61E-60	-3.38	0.00E+00
FTA_RS00625	pdpB1	WP_011457346.1	FPI, Pathogenicity determinant protein B1	-1.63	1.99E-30	-3.29	3.12E-121	-4.92	0.00E+00
FTA_RS05930	pdpB2	WP_011457346.1	FPI, Pathogenicity determinant protein B2	-1.65	1.69E-30	-3.28	1.22E-118	-4.93	0.00E+00
FTA_RS00620	iglE1	WP_003018235.1	FPI, Intracellular growth locus E1	-1.08	2.16E-02	-3.32	2.11E-13	-4.4	0.00E+00
FTA_RS05925	iglE2	WP_003018235.1	FPI, Intracellular growth locus E2	-1.56	2.38E-04	-2.78	2.48E-11	-4.34	0.00E+00
FTA_RS00615	vgrG1	WP_003016192.1	FPI, Valine-glycine repeat protein G1	-1.39	9.82E-05	-2.82	6.70E-16	-4.21	0.00E+00
FTA_RS05920	vgrG2	WP_003016192.1	FPI, Valine-glycine repeat protein G2	NA	NA	-3.75	1.14E-19	-4.15	0.00E+00
FTA_RS00610	iglF1	WP_003022130.1	FPI, Intracellular growth locus F1	-1.79	1.96E-13	-1.81	6.31E-14	-3.6	0.00E+00
FTA_RS05915	iglF2	WP_003022130.1	FPI, Intracellular growth locus F2	-1.86	4.09E-17	-1.68	2.02E-14	-3.55	0.00E+00
FTA_RS00605	iglG1	WP_003016185.1	FPI, Intracellular growth locus G1	NA	NA	-4.41	1.50E-35	-4.25	0.00E+00
FTA_RS05910	iglG2	WP_003016185.1	FPI, Intracellular growth locus G2	NA	NA	-4.36	3.14E-36	-4.24	0.00E+00
FTA_RS00600	iglH1	WP_003016182.1	FPI, Intracellular growth locus H1	-0.53	1.15E-02	-3.03	2.48E-50	-3.56	0.00E+00
FTA_RS05905	iglH2	WP_003016182.1	FPI, Intracellular growth locus H2	NA	NA	-3.17	5.09E-44	-3.6	0.00E+00
FTA_RS00590	iglI1	WP_010031639.1	FPI, Intracellular growth locus I1	-0.62	3.98E-02	-3.39	6.87E-32	-4.01	0.00E+00
FTA_RS05895	iglI2	WP_010031639.1	FPI, Intracellular growth locus I2	-0.87	9.32E-05	-3.11	1.40E-46	-3.98	0.00E+00
FTA_RS00585	iglJ1	WP_003016175.1	FPI, Intracellular growth locus J1	-0.77	4.10E-02	-2.72	8.27E-14	-3.5	0.00E+00
FTA_RS05890	iglJ2	WP_003016175.1	FPI, Intracellular growth locus J2	-0.86	1.82E-02	-2.61	9.83E-14	-3.47	0.00E+00
FTA_RS00580	pdpC1	WP_010031640.1	FPI, Pathogenicity determinant protein C1	-1.58	3.14E-51	-3.78	5.68E-289	-5.36	0.00E+00
FTA_RS05885	pdpC2	WP_010031640.1	FPI, Pathogenicity determinant protein C2	-1.64	1.75E-61	-3.72	0.00E+00	-5.36	0.00E+00
FTA_RS00575	pdpE1	WP_010031641.1	FPI, Pathogenicity determinant protein E1	NA	NA	-3.47	4.41E-28	-3.99	0.00E+00
FTA_RS05880	pdpE2	WP_010031641.1	FPI, Pathogenicity determinant protein E2	NA	NA	-4.09	2.35E-30	-4.06	0.00E+00
FTA_RS00570	iglD1	WP_010031642.1	FPI, Intracellular growth locus D1	1.22	9.18E-17	-5.97	0.00E+00	-4.75	0.00E+00
FTA_RS05875	iglD2	WP_010031642.1	FPI, Intracellular growth locus D2	1.4	9.98E-16	-6.15	8.68E-280	-4.75	0.00E+00
FTA_RS00565	iglC1	WP_003016165.1	FPI, Intracellular growth locus C1	2.2	1.98E-23	-8.46	0.00E+00	-6.26	0.00E+00
FTA_RS05870	iglC2	WP_003016165.1	FPI, Intracellular growth locus C2	2.2	6.73E-17	-8.46	5.12E-233	-6.27	0.00E+00
FTA_RS00560	iglB1	WP_010031646.1	FPI, Intracellular growth locus B1	1.68	1.42E-46	-6.82	0.00E+00	-5.14	0.00E+00
FTA_RS05865	iglB2	WP_010031646.1	FPI, Intracellular growth locus B2	1.94	9.33E-53	-7.09	0.00E+00	-5.14	0.00E+00
FTA_RS00555	iglA1	WP_003016161.1	FPI, Intracellular growth locus A1	2.49	2.22E-26	-6.4	2.64E-167	-3.91	0.00E+00
FTA_RS05860	iglA2	WP_003016161.1	FPI, Intracellular growth locus A2	2.92	5.92E-23	-6.84	4.02E-120	-3.92	0.00E+00

MglA is a crucial transcriptional regulator of *Ft* that regulates a variety of genes, particularly those within the FPI, and is required for *Ftn* replication in host cells and adaptation to oxidative stress (Baron and Nano, 1998; Bönquist et al., 2008). We found a transcriptional upregulation of *mglA* in tick cells (LFC = 1.59) compared to the macrophage environment. The heterodimer MglA/Stringent Starvation Protein A (SspA) also binds to the RNA-polymerase (Brotcke et al., 2006). *sspA* shows a similar regulation as *mglA* in *Fth* in tick and macrophage environment (LFC = 1.55).

PigR, a *Francisella* virulence factor (FevR) ortholog, is crucial for the expression of FPI genes within the MglA/SspA regulon (Brotcke and Monack, 2008; Charity et al., 2009). The expression of FevR is upregulated by MglA, and the combined effect of FevR and MglA further enhances the upregulation of the expression of virulence genes (Spidlova et al., 2020). The expression of PigR is also upregulated in the comparison between ticks and macrophages (LFC = 2.14).

The regulator OxyR (Michán et al., 1999), involved in oxidative stress response and controlling *ahpS*, *katG* and superoxide dismutase (*sodB*) gene expression (Lindgren et al., 2007; Ma et al., 2016), is also upregulated (LFC = 1.38). Deletion of OxyR results in a reduced ability of macrophages to grow (Ma et al., 2016). The RNA-binding protein Hfq, known for its involvement in sRNA-mRNA interactions, plays a crucial role in bacterial responses to various environmental conditions such as iron limitation, oxidative stress, anaerobic conditions and glucose starvation (Gottesman and Storz, 2011). Hfq regulates many genes, including the FPI genes, and has a particular effect on the pathogenicity determinant protein (*pdp*) operon. In most instances, Hfq acts as a negative regulator, as shown by transcriptome studies in *Ft* and reproduced in this study (*hfq*: LFC = 2.35, *pdpB*: LFC = 1.63, *pdp1*: LFC = 1.58, *pdp2*: LFC = 1.62).

Bacteria possess small basic regulatory proteins known as histone-like proteins or nucleoid-associated proteins, which are similar to eukaryotic histones (Stojkova et al., 2018). Like their eukaryotic counterparts, these proteins bind to DNA and influence gene expression by bending the DNA. Histone-like factor U (HU) is highly conserved across bacterial species and plays an essential role in several cellular functions. Disruption or dysfunction of these proteins could affect basic metabolic pathways and potentially attenuate the virulence of pathogenic bacteria (Weintraub and Van Lente, 1974; Dillon and Dorman, 2010). Recent research has highlighted the importance of the HU protein for intracellular growth and virulence of *Fth* (Stojkova et al., 2018). HU is encoded by the *hupB* gene, forms homodimers in *Francisella* and plays a role in processes such as recombination, replication, transcription and influences DNA shape (Azam and Ishihama, 1999). As a transcription factor, HU regulates important genes such as *pigR* and FPI genes. Interestingly, *hupB* was found to be upregulated in ticks compared to the macrophage environment (LFC = 1.73).

The AraC protein, which belongs to the AraC/XylS family, has the highly conserved helix-turn-helix (HTH) DNA-binding domain, which is essential for its function as a transcription factor. In *Fth* LVS, a mutant strain lacking the AraC-encoding gene, the role of the protein in regulating the response to oxidative stress has been demonstrated (Marghani, 2019). The activation of AraC occurs specifically under oxidative stress conditions, where it downregulates the five FPI genes *pdpE*, *pdpC*, *iglJ*, *iglI* and *dotU*. In addition, AraC plays a critical role in regulating the expression of key components of the tricarboxylic acid (TCA) cycle, including the downregulation of pyruvate dehydrogenase and oxoglutarate dehydrogenase. Although we did not detect any transcripts of *araC* in our samples, the three FPI genes regulated by the protein are downregulated (*pdp* C: LFC = -1.58e, *iglJ1*: LFC = -0.77, *iglJ2*: LFC = -0.86, *iglI1*: LFC = -0.62, *iglI2*: LFC = -0.82).

Iron uptake regulator (Fur) is a homotetrameric protein capable of binding specific DNA sequences by cleaving into two dimers. It regulates iron levels and contributes to metal homeostasis in bacterial cells (Pérard et al., 2018). Fur functions primarily as an Fe2+-dependent transcriptional repressor that downregulates genes associated with the tricarboxylic acid cycle (Troxell et al., 2011). Iron levels in host cells influence intracellular replication and virulence of *Fth* (Troxell and Hassan, 2013). Our study revealed an upregulation of *fur* in the environment of tick cells compared to macrophages (LFC = 1.11). This observed differential expression may indicate a nuanced adaptive response of *Francisella* to variations in iron availability.

The IclR family of transcription factors is ubiquitous in bacterial species. It is characterized by the presence of a helix-turn-helix domain that facilitates DNA binding (Molina-Henares et al., 2006). While it plays a critical role in the virulence of *Ftn* in a mouse model, IclR is dispensable for the full virulence of *Ftt* or *Fth*, although it is highly conserved in several *Francisella* species (Robertson Fuller et al., 2009). In particular, the observed upregulation of the *iclR* gene in the tick environment compared to macrophages (*iclR*: LFC = 1.11) raises questions about its regulatory role in different hosts.

RNA polymerase and sigma factors ensure specific recognition of the promoter. The RNA polymerase of *Francisella* consists of two α-subunits (encoded by *rpoA1* and *rpoA2*), β-, β′- and ω-subunits (encoded by *rpoB*, *rpoC* and *rpoZ)* (Charity et al., 2007). In addition, *Francisella* has two sigma factors: the major sigma factor σ70 (encoded by the *rpoD* gene) and a heat shock protein homolog of theσ32 family, encoded by the *rpoH* gene (Charity et al., 2007; Grall et al., 2009). The genes encoding the subunits of the RNA polymerase are all upregulated (*rpoA*: LFC = 1.61, *rpoB*: LFC = 2.92, *rpoC*: LFC = 2.18, *rpoZ*: LFC = 1.90, *rpoD*: LFC = 0.14e, *rpoH*: LFC = 0.24) in ticks compared to macrophages.

Two-component systems (TCSs) typically consist of a membrane-bound sensor histidine kinase and a DNA-binding response regulator that allow signal transduction and an adequate response of gene expression to changing environmental conditions. When an outside signal is detected, the sensor kinase is phosphorylated and the response regulator is activated and functions as a transcription factor (Stock et al., 2000; Van Hoek et al., 2019). The presence of TCS components varies between *Francisella* species and subspecies. *Fth* contains a response regulator (PmrA) and an orphan sensor kinase (QseC). We observed upregulation of *pmrA*/*qseC* genes in *Fth* infected in ticks compared to macrophages (*pmrA*/*qseC*: LFC = 2.37).

### 3.4 Comparison of the differentially expressed genes and proteins in the basal groups B.6 and B.12

**Figure 4:**
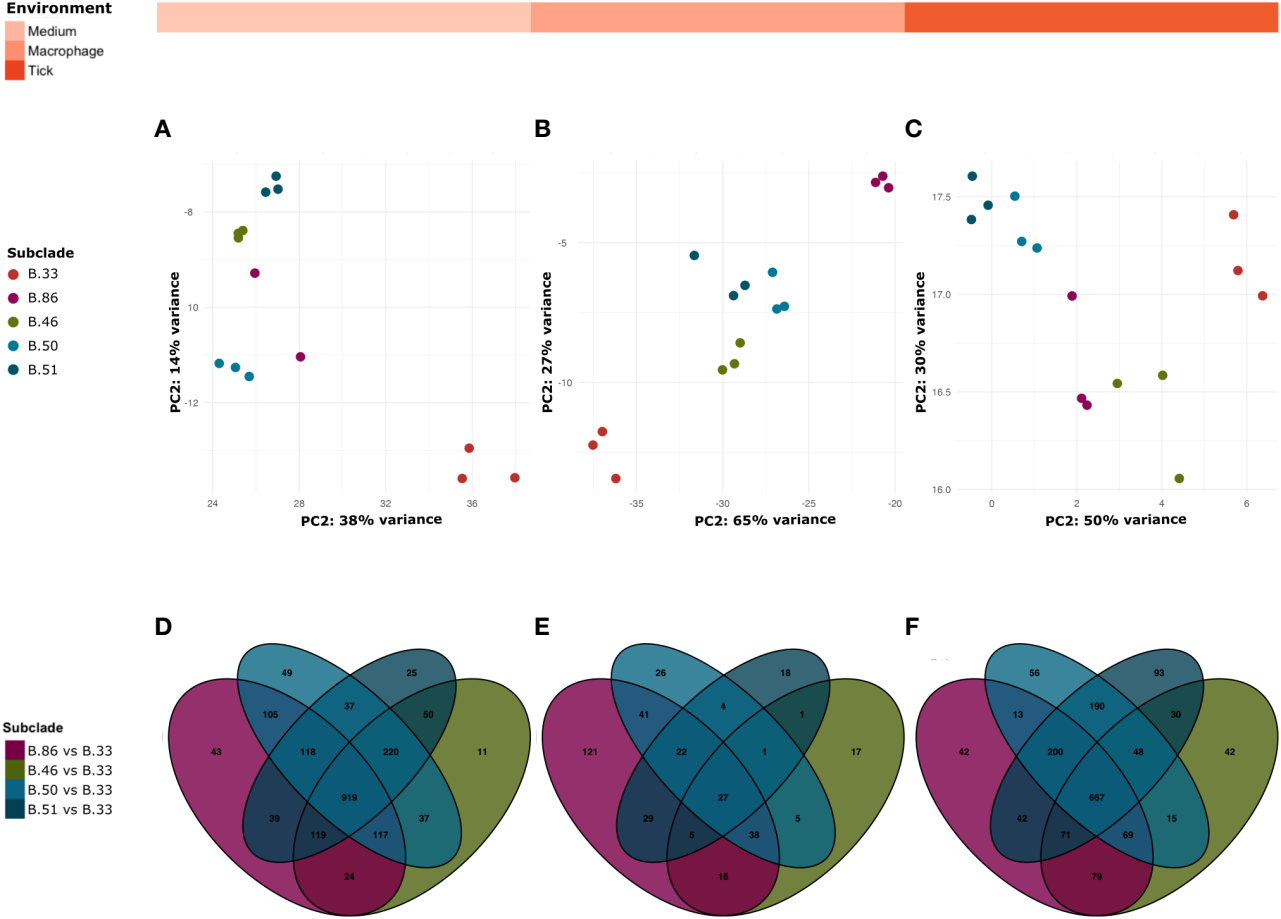
The strains of the B.6 group (B.86, B.46, B.50, B.51) compared to the B.12 strain B.33 showing that the majority of DEGs are shared by the B.6 strains in all environments. Principal component analysis (PCA) of the different genotypes B.33, B.86, B.46, B.50, B.51 in triplicate used for RNA sequencing in the medium **(A)**, macrophage **(B)** and tick **(C)** environments. All samples represent consistency of data between replicates. Venn diagram of DEGs in medium **(D)**, macrophage **(E)** and tick cells **(F)** showing the number of common and unique genes between the different subgroups B.86 vs B.33, B.46 vs B.33, B.50 vs B.33 and B.51 vs B.33 (padj < 0.05, absolute LFC > 1).

RNA-sequencing analyses showed that whole genome expression profiles from biological replicates corresponding to different genotypes belonging to different subclades B.33, B.86, B.46, B.51 and B.50 were clustered in all environments, suggesting that *Fth* changes its transcriptional profile according to its clade (**Figures 4A-C**).

The strains belonging to the B.6 clade (B.86, B.46, B.50, B.51) compared to the strain B.33 of clade B.12 revealed that the majority of the DEGs is shared by the B.6 strains in all environments. As shown in the Venn diagrams in **Figure 4**, 919 genes are identified as identically differentially expressed in the medium (D), 27 in the macrophage (E) and 669 in the tick cell (F) environments.

Furthermore, we performed a KEGG enrichment analysis of the different genotypes belonging to the B.6 clades compared to the genotype of B.33 belonging to the B.12 clade. In the medium environment, several enriched metabolic pathways involved in energy production were downregulated. In contrast, metabolic pathways such as “ribosome” and “metabolic pathways” were upregulated in the macrophage and tick environments (**Supplementary Table 2**).

**Figure 5:**
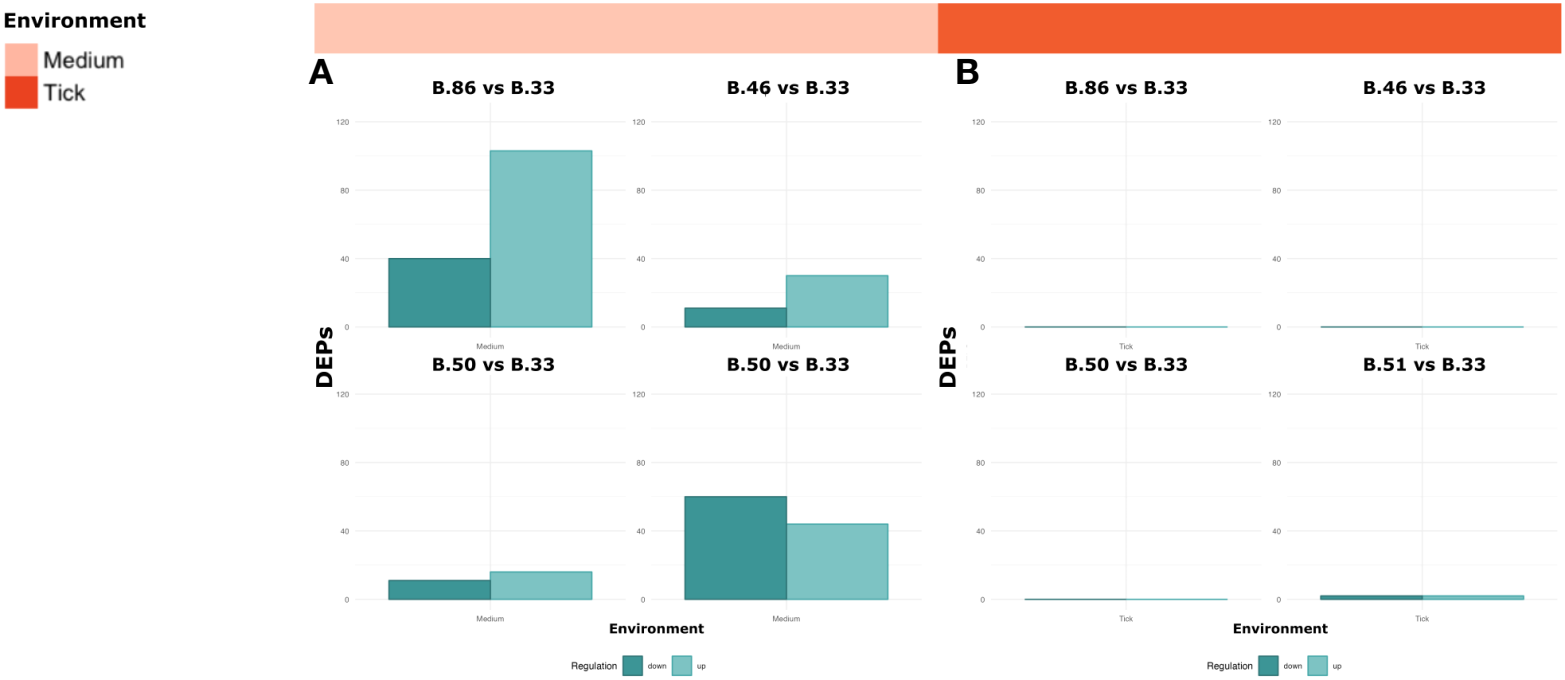
The number of DEPs varied between the different environments comparing the strains belonging to the B.6 clade (B.86, B.46, B.50 and B.51) to the strain B.33 (B.12 clade). Bar charts of DEPs in medium **(A)** and tick **(B)** environments for the comparisons B.86, B.46, B.50, B.51 vs B.33 (padj > 0.05, absolute LFC > 1).

The number of differentially expressed proteins (DEPs) that we were able to measure varied between the different environments (**Figure 5**). In the medium, we measured approximately 10% more DEPs (padj < 0.05, absolute LFC > 1) than in tick environment for the comparison B.51 vs B.33. No differentially expressed proteins could be measured in the tick environment for the comparisons B.86, B.46 and B.50 vs B.33.

**Figure 6:**
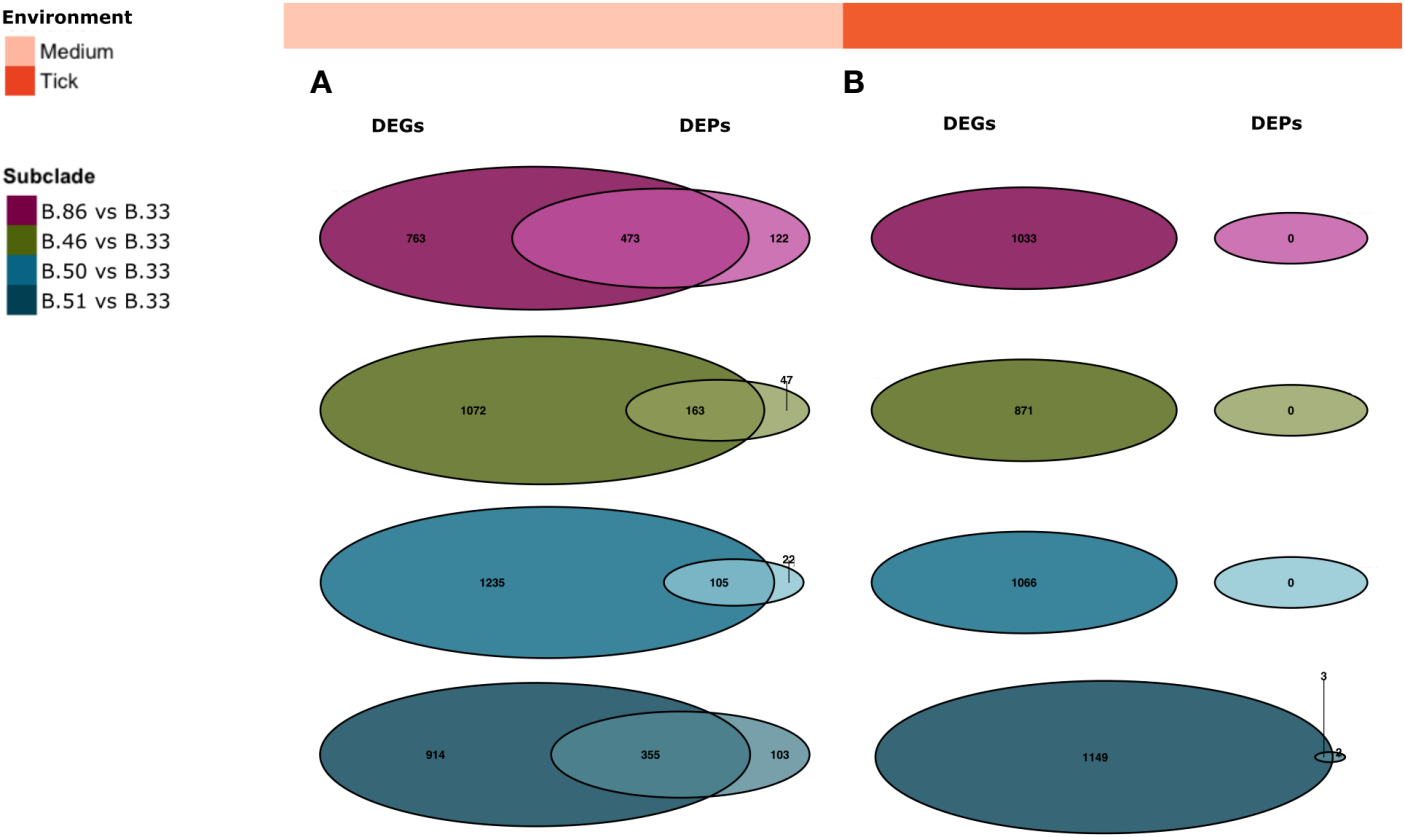
*Comparison of DEGs translating for the same proteins as the DEPs.* Venn diagram of B.86 vs B.33, B.46 vs B.33, B.50 vs B.33 and B.51 vs B.33 for medium **(A)** and tick cell **(B)** showing the number of common and unique proteins DEGs (left) vs DEPs (right) (padj < 0.05, absolute LFC > 1).

To determine whether we could measure the same differentially expressed proteins in the transcriptome and proteome data, we compared the two data sets in **Figure 6**. The DEGs translating for the same proteins as the DEPs were compared and are shown in the Venn plots for B.86 vs B.33, B.46 vs B.33, B.50 vs B.33 and B.51 vs B.33 in medium and tick environments (padj < 0.05, absolute LFC > 1).

In the medium, 219, 162, 106 and 355 similarly expressed proteins were identified for the comparison B.86 vs B.33, B.46 vs B.33, B.50 vs B.33 and B.51 vs B.33. In the tick environment, only one identical protein could be identified in B.51 vs B.33 in both data sets.

## 4 Discussion

Increasing numbers of both, ticks and *Ft* infections in humans, emphasize the need to better understand the life cycle of this pathogen. It is likely that during the transition between mammalian hosts and tick vectors, *Ft* undergoes significant changes, including substantial alterations in gene and protein expression profiles. Laboratory experiments have confirmed the persistence of *Ft* in ticks, supporting their role as a potential reservoir in the environment (Mani et al., 2015; Tully and Huntley, 2020). The transstadial transmission and replication within ticks further establishes them as both a reservoir and a vessel for the amplification of *Ft* (Coburn et al., 2015). Rabbits are primary sources of human infections in many tularemia endemic areas. However, these animals often succumb rapidly from tularemia and thus might not be considered long-term reservoir of *F. tularensis* (Brown et al., 2015; Sonenshine, 2018). Limited information is available on the genes and proteins of *Ft* required to infect, persist, replicate in ticks and transmit to mammalian hosts. To the best of our knowledge, only one investigation has examined the ability of a *Ft* Δ*purMCD* mutant to infect and replicate in ticks (Zellner and Huntley, 2019). Despite that the Δ*purMCD* strain was avirulent in mice, it showed successful colonization of the tick *Dermator variabilis*. However, during the molt to the adult stage, it failed to persist in these ticks. This finding suggests that purine synthesis is essential for *Ft* transstadial transmission in ticks, similar to some biosynthetic pathways that are critical for infection in mammals. These proteins play a crucial role in fine-tuning the adaptation to changing environmental conditions. As an important human pathogen, *Francisella* has to cope with the challenges posed by the immune system. Consequently, these regulatory proteins may be key elements in the immune evasion of *Francisella*. To fully understand the pathogenesis of *Francisella* and to formulate effective strategies to counteract *Ft* infections, a detailed investigation of these pathways is required.

In the three environments, medium, macrophages and tick cells, we detected transcriptional changes in gene expression of all investigated *Fth* genotypes. Two distinct significantly differentially expressed gene clusters were identified. The first cluster consisted of upregulated genes involved in the protein synthesis pathway. These genes were upregulated in macrophages and similarly expressed in the tick environment. The second cluster contains downregulated genes of the metabolic pathway and is downregulated in macrophages and similarly expressed in the tick environment (**Figure 1**). Many intracellular bacteria are exposed to adverse conditions within the vacuole during infection. These include low pH, free radicals and nutrient deprivation. Although *Francisella* replicates in the host cell cytosol, which is considered to be more permissive than the phagosomal compartment, this environment remains more stressful than the standard growth conditions of the medium. The downregulation of the *Ft* pathway in macrophages compared to standard media conditions could be due to intracellular stress factors affecting the metabolism of the pathogen. Compared to macrophages, *Fth* in ticks appears to be metabolically similarly active than in the medium, which could be explained by a combination of a better suited environment to the nutritional needs of *Fth* in ticks and/or fewer intracellular stress factors. (**Figure 1**). The detailed understanding of the metabolic pathways and specific nutrient utilization during intracellular replication of *Ft* remains limited. Bacteria that replicate in the cytosol of the host cell make use of the products and intermediates generated by the metabolic processes of the host, such as glycolysis and amino acid biosynthesis. However, their immediate accessibility by intracellular pathogens is hindered by their storage in complex structures, and the precise concentration of these products within infected cells remains elusive (Brunton et al., 2015).

This study revealed distinct transcriptomic changes between tick cell and macrophage infection. In particular, upregulation of several pathways related to carbon metabolism was observed. In the absence of glucose, it is plausible that *Francisella* relies predominantly on available carbon sources, such as pyruvate or tricarboxylic acid (TCA) cycle intermediates, in conjunction with amino acids that serve as intracellular carbon and nitrogen sources (Radlinski et al., 2018). A shift to non-glucose substrates may be suspected by the observed upregulation of genes of the TCA cycle. Furthermore, the pathway involved in the transport of proteins across the membrane, the bacterial secretion system pathway.

Ticks are strictly hematophagous, i.e. they feed exclusively on blood throughout their development. Although blood is rich in many nutrients, it is relatively poor in others, such as B-vitamins. A previous study found that vitamin B is essential for the development of the soft tick *Ornithodoros moubata* and that *Francisella* acts as a supplier of vitamin B (Duron et al., 2018). Our results indicate the involvement of *Francisella* in *Ixodes ricinus* tick nutrition by differentially regulating the bacterial biotin synthesis pathway (**Figure 2**).

Bacteria often use TCS to sense and respond to their changing environment. These systems are widespread in bacteria. They consist of a histidine sensor kinase, which is sensitive to environmental cues, and a response regulator, which triggers specific cellular responses (Stock et al., 2003). In contrast to many other bacteria, *Francisella* has a limited number of TCSs in its genome. We found that the genes coding for the PmrA and QseC, *pmrA/qseC*, are upregulated in the tick cells. It has been proposed that once phosphorylated, PmrA binds to the promoters of regulated genes and recruits free or RNA-polymerase-bound MglA and SspA to initiate the transcription of the FPI genes, including *pdpABCDE*, *iglABCDEFGHIJ* and *vgrG* (Bell et al., 2010). In the tick environment, both *mglA* and *sspA* were found to be upregulated compared to growth in macrophage. Furthermore, *iglABCD* were upregulated. However, *pdpBC* were downregulated. *iglFG* and *pdpAE* were not found to be differentially expressed. Thus, an initiation of many, but not all of the FPI genes could be observed during tick infection compared to macrophage infection. Most FPI genes are involved in Type 6 Secretion and those essential for Type 6 Secretion were upregulated (with the exception of *pdpB*) (Brodmann et al., 2017). To understand the pathogenesis of *Ft* in this specific environment, a comprehensive insight into its regulation is crucial.

The RNA polymerase and sigma factors required to transcribe genes are slightly upregulated in the tick environment. It has been shown previously that these factors are required for the intracellular growth and full virulence of the *Ftn* (Gray et al., 2002). The Fur protein controls the transcription of bacterial genes involved in iron acquisition. Fur gene expression is upregulated in the tick environment compared to macrophages, potentially pointing towards iron limitation in ticks (**Figure 3**).

In Europe the basal clades B.6 and B.12 of *Fth* are present. In Switzerland, the majority of strains belong to B.6. Different geographical distributions and host preferences were observed for the different B.6 subclades. The most common subclade, B.45, is distributed throughout northern Switzerland and has been found in humans, animals and ticks (Dwibedi et al., 2016; Wittwer et al., 2018; Schütz et al., 2023). Moreover, basal clades B.6 and B.12 have been reported to show differences in pathogenicity in lagomorphs. Whole genome expression profiles from biological replicates clustered according to different genotypes belonging to different subclades B.33, B.86, B.46, B.51 and B.50 in all environments, suggesting that *Fth* alters its transcriptional profile according to genotype. Comparison of the strains belonging to the B.6 clade with the B.12 clade showed that the majority of genes are shared between the B.6 clades (**Figure 4**). This may indicate that only a few expressed proteins contribute to the described phylogenetic and functional differences.

We only identified differentially expressed genes or proteins between B.6 and B.12 isolates when cultured in medium, but not in tick cells or macrophages (**Figure 5**). Another methodological limitation is the different growth of *Fth* in the different host environments. Those differences in RNA-seq library sizes complicated the comparison between conditions. Additionally, due to the small number of bacterial genes/proteins compared to the host, the measurement of intracellular bacterial genes, but especially proteins is challenging. In this study, this was also a challenge. Further development of more sensitive protocols will be necessary in the future. Tick cells and macrophages were cultured in different media, based on their requirements. Different media might also influence the transcriptomes and proteomes of intracellular bacteria. Other limitations of this study include using cell lines that may not mimic the complex processes of tick infection. Ticks have an innate immune system that includes both cellular and humoral components to fight infections (Ayllón et al., 2013; Sunyakumthorn et al., 2013; Ayllón et al., 2015). Consequently, infections of live ticks may provide more revealing information about possible adaptive differences of individual subclades to the tick vector and the resulting host response.

## 5 Conclusion

This study reports changes in the *Fth* transcriptome in the tick environment in comparison to mammalian macrophages. We were able to characterized key pathway adaptations to the different metabolic demands of the tick cell environment. We also found that the tick and macrophage environments differentially regulate known transcription factors and their targets involved in virulence. This may have implications for our understanding of the transmission of *Francisella* between the mammalian host and the tick vector.

## Data availability statement

The datasets presented in this study can be found in online repositories. The names of the repository/repositories and accession number(s) can be found below: https://www.ncbi.nlm.nih.gov/, PRJNA1050617.

## Ethics statement

Ethical approval was not required for the studies on animals in accordance with the local legislation and institutional requirements because only commercially available established cell lines were used.

## Author contributions

SS: Formal analysis, Investigation, Visualization, Writing – original draft, Data curation. MB: Formal analysis, Writing – review & editing. NL: Writing – review & editing. MM: Writing – review & editing. MW: Conceptualization, Funding acquisition, Project administration, Writing – review & editing. RB: Conceptualization, Funding acquisition, Project administration, Supervision, Writing – review & editing.

## Supplementary material

Supplementary Table 1 Fth strains used in this study.

Supplementary Table 2 Identified KEGG pathways by GSEA analysis (padj < 0.05, absolute LFC > 1) of DEGs in B.86 vs B.33, B.46 vs B.33, B.50 vs B.33, B.51 vs B.33 in medium, tick and macrophage environments; LFC: Log2 fold change; padj: adjusted p-value.
